# Loss of Retinal Function and Pigment Epithelium Changes in a Patient with Common Variable Immunodeficiency

**DOI:** 10.1155/2012/967561

**Published:** 2012-09-29

**Authors:** Jakob Halborg, Torben L. Sørensen

**Affiliations:** ^1^Department of Ophthalmology, Copenhagen University Hospital Roskilde, 4000 Roskilde, Denmark; ^2^Faculty of Health Sciences, University of Copenhagen, 2200 Copenhagen, Denmark

## Abstract

Common variable immunodeficiency (CVID) has only scarcely been associated with ocular symptoms and rarely with retinal disease. In this case we describe a patient with distinct morphological and functional alterations in the retina. The patient presents with characteristic changes in retinal pigment epithelium, autofluorescence, and electrophysiology.

## 1. Introduction

Common variable immunodeficiency (CVID) is a group of heterogenic disorders affecting both children and adults [1]. CVID is characterized by disturbances in both the innate and the adaptive immune system resulting in recurrent infections, lymphoproliferative disease, granulomatous disease, and autoimmunity affecting multiple organs. The patients have hypogammaglobulinemia with poor antibody production and responses. Eye involvement in CVID has only been scarcely described. One report described retinal vasculitis in a case series of 3 patients affected by CVID in childhood [2], and two case reports have described the occurrence of uveitis in a child and in a young adult [3]. Another adult patient showed signs of keratoconjunctivitis as an onset manifestation of CVID [4]. One case report has described choroidal changes in patients with CVID [5]. We describe a patient with CVID who developed loss of retinal function and distinct morphological changes at the retinal pigment epithelium (RPE) level. To our knowledge this has not been observed before.

## 2. Case Presentation

Our patient is a 63-year-old female with a long medical history of recurrent infections and problems with the gastrointestinal system. Blood tests revealed very low levels of immunoglobulin (Ig)-G, and a diagnosis of CVID was made about ten years ago. She now receives treatment with intravenous immunoglobulins. 

We first saw the patient in 2007 because of an itching and burning sensation in both eyes. She had quite severe keratoconjunctivitis sicca syndrome, but visual acuity was normal, and the rest of the ophthalmological examination was unremarkable. 

In 2011 she complained of diminished vision on both eyes and photophobia. Best corrected visual acuity had dropped to 0.3 on both eyes. She had developed concentric visual fields defects to approximately 10–30° on both eyes assessd with Goldman visual field testing and diminished colour vision using Farnsworth Panel D-15. Magnetic Resonance Imaging (MRI) of the cerebrum was normal apart from small areas with gliosis. Full-field electroretinogram (ERG) showed diffuse amplitude reduction and delayed implicit time. Multifocal ERG revealed markedly reduced amplitudes in the central area. 

**Figure 1:**
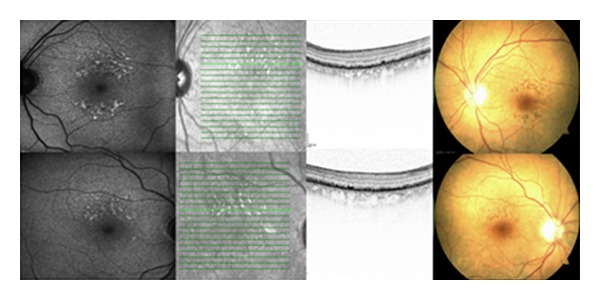
Autofluorescent imaging, SD-OCT and colour fundus photography of both eyes. Note the marked increase of autofluorescense and changes at the RPE level on OCT. (Light green denotes scanning position).

Funduscopy revealed retinal changes in both eyes (Figure 1). Autofluorescense imaging showed a diffuse pattern of increased autofluorescence around the entire macula with a marked increase of autofluorescence around the fovea (Figure 1). Spectral-Domain Optical Coherence Tomography OCT (SD-OCT), (Heidelberg Engineering, Heidelberg, Germany) showed marked RPE changes with accumulation of material in the areas of increased autofluorescense. The RPE changes were characterized by an increased thickness of the RPE band on SD-OCT and RPE thickening was predominantly localized around the area of pigment derangement observed on funduscopy (Figure 1). There were no areas of separation between Bruch's membrane and RPE and no signs of drusen either on fundoscopy or on SD-OCT. Fluorescein angiography revealed hypofluorescence related to the RPE changes but no leakage or any vascular changes were observed.

Observation during one year showed gradual increase in the RPE changes.

## 3. Discussion

Ocular involvement in CVID is rarely described, and no previous reports have described loss of retinal function nor have suggested an involvement at the RPE level in CVID. This patient has two forms of ocular involvement. One is keratoconjunctivitis sicca, which has been previously described in CVID [4] but our patient also has diffuse loss of retinal function and marked symmetrical changes in the RPE resulting in increased autofluorescense patterns and changes of the RPE on SD-OCT. Even though there are some resemblances to age-related macular degeneration (AMD) some hallmarks are missing such as drusen. Furthermore AMD rarely presents with circumscribed lesions in the RPE around the fovea. One could consider whether this could be Pattern Dystrophy; however these lesions are more confluent, predominantly butterfly shaped and yellowish, whereas the changes in this patient are more pigmented. Another possibility could be Stargardt's disease but some of the such as hallmarks characteristics of Stargardts disease, dark choroid, “fish tails,” are missing and the age of onset is not typical for Stargardt's disease. Also the lesions in Stargardt's disease are, just as in Pattern Dystrophy; more yellowish rather than pigmented as seen in this patient. None of the known age-related macular changes produce loss of function, like the one which have been demonstrated in this patient. The patient never received any medication known to cause RPE changes. There were no signs of granulomatous choroidal involvement or uveitis, suggesting a local inflammatory component at the RPE level. 

This is the first description of RPE and retinal functional changes in a patient with CVID. A distinct causal relationship still needs to be established, and since simultaneous CVID and retinopathy only has been seen in this one patient our finding could simply be due to chance. However, this paper might draw attention to possible retinal findings in this rare disorder.
